# GERONTOLOGY DOCTORAL EDUCATION: RELEVANCE AND CHALLENGES AT UNIVERSITY OF MARYLAND, BALTIMORE AND BALTIMORE COUNTY

**DOI:** 10.1093/geroni/igae098.1619

**Published:** 2024-12-31

**Authors:** John Schumacher, Ann Gruber-Baldini

**Affiliations:** University of Maryland, Batlimore County (UMBC), University of Maryland, Batlimore County, Maryland, United States; University of Maryland School of Medicine, Baltimore, Maryland, United States

## Abstract

The joint University of Maryland, Baltimore County (UMBC) & Baltimore’s (UMB) Doctoral Program in Gerontology celebrated the milestone of its 50th graduate in 2024. Yet, like our peer doctoral programs, gerontology education remains undervalued in society and higher education. Recent ways we highlight our relevance include: 1) Launch of an Online Masters in Gerontology. Sustainability may lie in universities sharing enrollment across online courses; 2) Created Global Experiences i) Costa Rican Fellowship. External donor funded program for gerontology students and faculty for an extended, bi-directional scholarly exchange. ii) Global Aging Gerontology in Japan. Immersive interdisciplinary course held in Japan as a super-aging society; 3) Generative Artificial Intelligence (Gen AI) in Gerontology Education & Research. Training faculty and students to participate in an AI infused work environment. Challenges to doctoral education abound including: 1) Funding graduate research assistantships (GRAs); decreasing GRA allocations and both increasing cost and pressures to exclusively fund GRAs on external grants; 2) Engaging faculty effort to move beyond a voluntary, gerontology affiliate faculty model. Ever increasing demands on faculty time are leading to less service availability to gerontology programs. New faculty affiliate models are needed that may involve compensation and recognition for interdisciplinary faculty effort and revenue sources. Overall, degree programs in gerontology must continue to promote their relevance while meeting continuing challenges.

